# Morel-Lavallée Lesion in a Female Breast: A Case Report

**DOI:** 10.7759/cureus.42979

**Published:** 2023-08-05

**Authors:** Masayasu Takegawa, Satoshi Yoshimura, Nobuhiro Ikeda, Nobuhiro Miyamae, Yasuyuki Sumida

**Affiliations:** 1 Department of Plastic Surgery, Rakuwakai Otowa Hospital, Kyoto, JPN; 2 Department of Preventive Services, Kyoto University, Kyoto, JPN; 3 Department of Emergency Medicine, Rakuwakai Otowa Hospital, Kyoto, JPN

**Keywords:** woman in surgery, emergency medicine and trauma, female breast, closed degloving injury, morel–lavalée lesion

## Abstract

Morel-Lavalée lesions (MLLs) are caused by closed degloving injuries that mostly occur in the thigh, flank, and buttocks. We report a rare case of an MLL in the breast that was successfully treated with surgical removal in the acute phase. A 57-year-old woman sustained a breast injury from a seatbelt in a motor vehicle accident. Contrast-enhanced CT was performed, and the patient was diagnosed with an MLL in her breast. Surgical removal was performed after five days, and the patient was discharged six days postoperatively. No recurrence was observed, and the cosmetic results were good at six months postoperatively. Early detection and treatment of MLL in the female breast are critical to avoid recurrence and ensure good cosmetic outcomes.

## Introduction

Morel-Lavalée lesions (MLLs) were first reported by Morel-Lavalée in 1863. MLLs are characterized by hemolymphatic fluid collection between the subcutaneous tissue and deep fascia due to a closed degloving injury [1]. It predominantly occurs in the thigh, flank, and buttocks [2], and motor vehicle accidents are the most common cause [2,3].

A wide array of treatments has been documented for managing MLLs, including compression, needle aspiration drainage, drug injection through sclerotherapy, and surgical resection [1]. However, there are still no universally accepted guidelines for the treatment of this condition. Although MLLs have been extensively described in the literature [2,3], MLLs in the breast have not been reported yet. Selection of the appropriate treatment protocol remains largely contingent upon the clinician's discretion based on the patient's unique clinical profile. However, immediate surgical intervention may have to be considered in certain cases, given the risk of cosmetic deformities resulting from chronic MLLs. In this report, we present a rare case of MLL in the breast caused by a motor vehicle accident and describe the successful surgical treatments in the acute setting.

Written informed consent was obtained from the patient for the publication of this case report and any accompanying images.

## Case presentation

**Figure 1:**
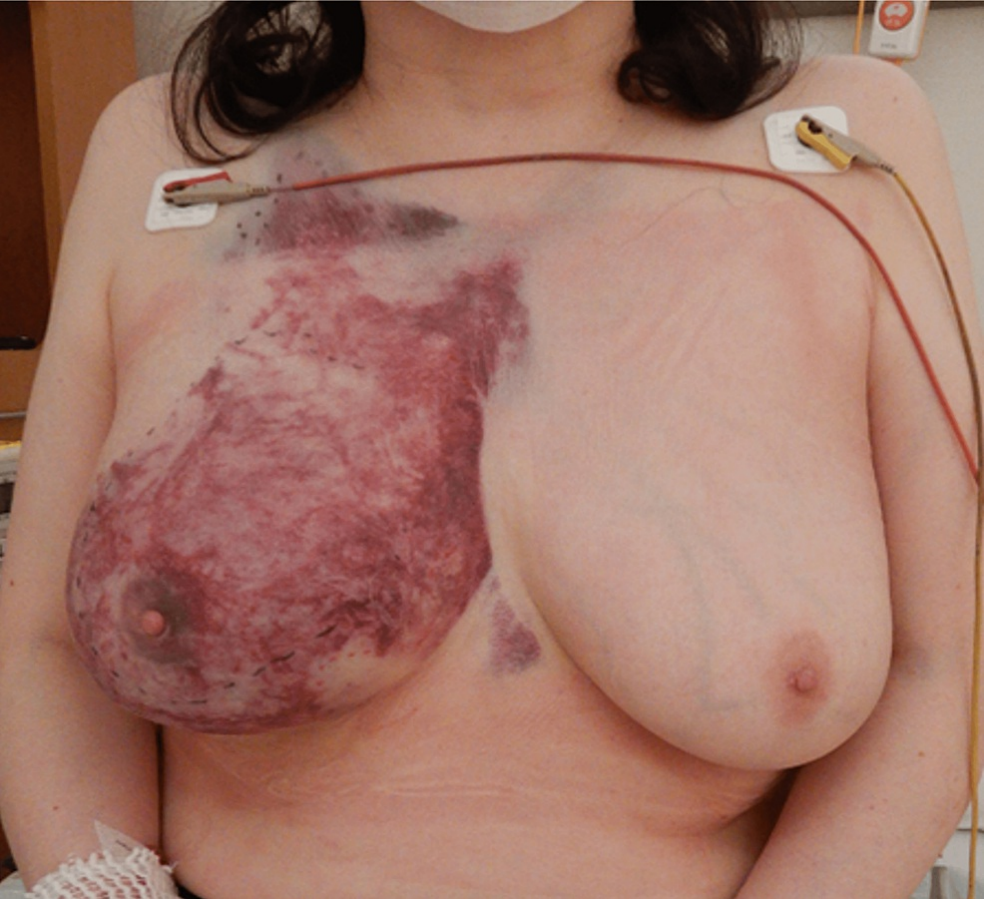
Image of the patient's breast the day after injury The right side had swelling and purpura extensively

The patient was a 57-year-old woman; she had been riding in her husband’s car that was hit by an oncoming car driven by a driver who had dozed off. She had been restrained by the seat belt and her car had airbags. When the driver’s side of the car collided, the patient remained in the passenger seat. The speed of both cars was unknown. Immediately after the accident, the patient experienced pain in her right anterior chest and was transported to our hospital. No major abnormalities were observed in the vital signs at the time of arrival, and the primary survey did not reveal any abnormalities either. In a secondary survey, we palpated an induration with painful subcutaneous hemorrhagic spots on the right anterior chest (Figure 1). Blood tests including complete blood count, chemistry, and coagulation were normal. No bleeding tendency was observed in the patients’ medical or drug histories.

**Figure 2:**
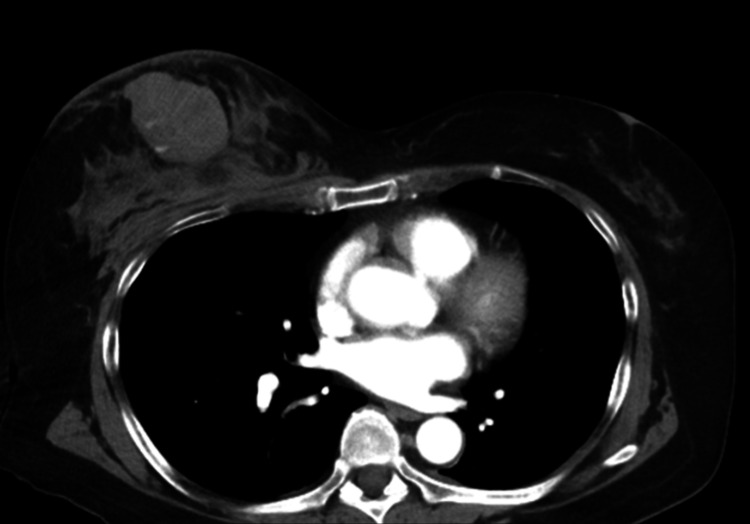
Contrast-enhanced CT Contrast-enhanced CT showing fluid collection with extravasation between the mammary gland and the pectoralis major muscle in the right anterior chest CT: computed tomography

Contrast-enhanced CT demonstrated a fluid collection with extravasation between the mammary gland and the pectoralis major muscle in the right anterior chest and bleeding from the penetrating branch of the intercostal artery or internal thoracic artery. Hematoma formation was suspected (Figure 2). No other fractures or organ damage were observed. The patient was diagnosed with MLL, and compression was applied to the right breast using gauze and a bandage.

**Figure 3:**
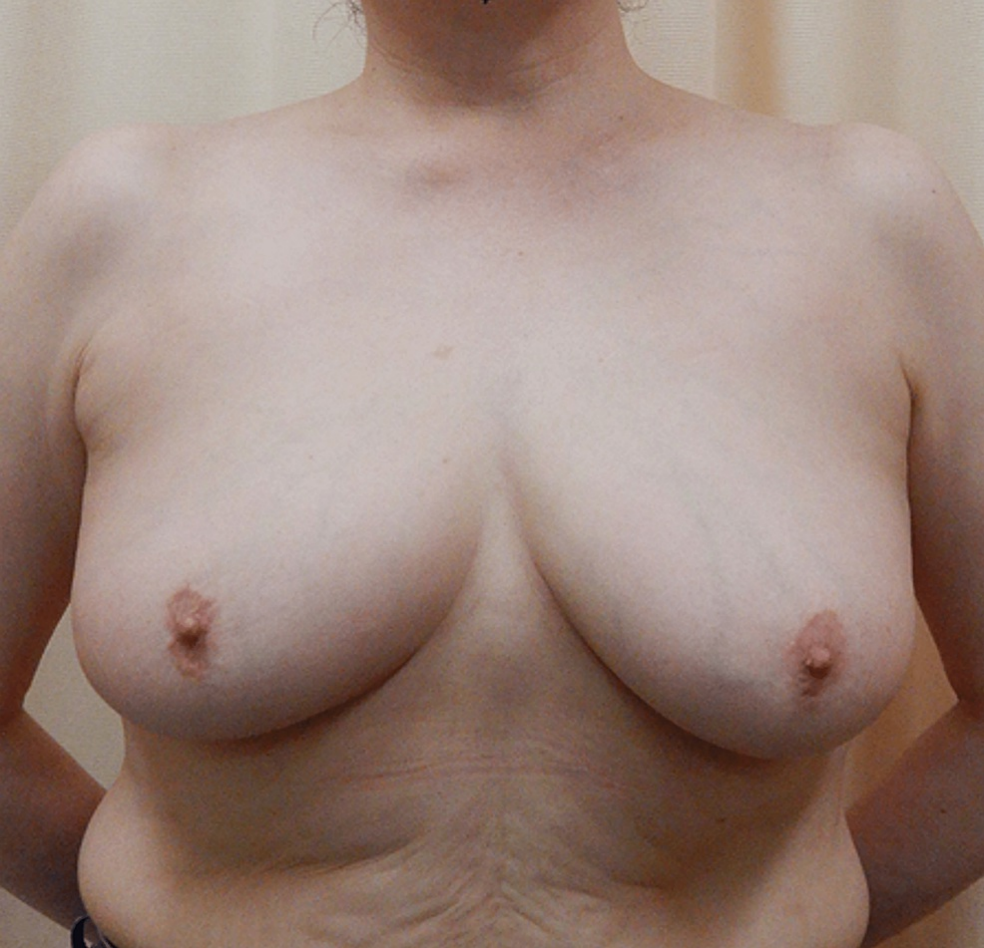
The breast after six months No recurrence was observed, and the cosmetic result was good

The patient underwent surgery five days later. The inframammary fold was incised, the pectoralis major fascia was detached, and finally, the hematoma was reached. It was removed and washed out, a closed continuous suction drain was placed, and the wound was sewn up. No postoperative complications were observed, and the drain was removed two days postoperatively. The patient was discharged six days postoperatively. At the six-month follow-up postoperatively, no recurrence was observed on subcutaneous ultrasonography, the operative scar was invisible, and there was no breast deformity (Figure 3).

## Discussion

MLLs were first reported by Morel-Lavalée in 1863, and motor vehicle accidents are their most common cause [2,3]. This soft tissue injury has been called a closed degloving injury, post-traumatic soft tissue cyst, Morel-Lavallée effusion, and chronic expanding hematoma (CEH) [4]. CEH was first reported by Reid et al. in 1980 as a hematoma that had grown for more than a month [5]. MLL is the concept of lesions focusing on the mechanism of injury, whereas CEH is the concept of lesions focusing on the course of the hematoma [6]. Therefore, trauma does not necessarily cause CEH. However, they are essentially identical.

An MLL results from a closed degloving injury that causes acute separation of the superficial epidermal and hypodermal layers from the deeper fascia [2]. Our patient experienced a large shear force between the trunk and breasts with high mobility. MLLs are reported to occur predominantly in the lower limbs (64%), with cases in the chest accounting for only 1% of all cases [3]. However, many female patients may have unrecognized MLLs in the breast because the lesion cannot be detected without an imaging examination. Therefore, physicians can diagnose swellings or contusions without follow-up examinations. Additionally, the more widespread the seatbelt legislation, the more severe the patients' injury will be, as in our case. Some cases of seatbelt injuries in females have been attributed to MLL in the breasts [7,8].

The MLL may also contain blood, lymph, or debris. Depending on the age of the internal blood products, they exhibit various imaging features [4]. Mellado and Bencardino classified MLL into six types based on their chronicity, tissue composition, and lesion appearance on MRI, as follows - type I: seroma; type II: subacute hematoma; type III: chronic organizing hematoma; type IV: perifascial dissection with closed fatty laceration; type V: perifascial pseudonodular lesion; and type VI: infected lesion with or without sinus tract formation, internal septations, and a thick enhancing capsule [9]. Ultrasonography can also produce images that reflect these characteristics to some extent [4]. Because of its simplicity, it is an important diagnostic tool in daily clinical practice.

Because MLLs are very rare, no definitive treatment method has been established, and conservative treatments such as compression, drainage by needle aspiration, sclerotherapy via drug injection such as doxycycline, and surgical resection have been described in the literature [1]. Although all of these methods seem to have a certain therapeutic effect, the need for follow-up should be emphasized when conservative treatment is administered or if the lesion is missed. If MLLs are not absorbed and become chronic, they form a capsule that is difficult to treat [2]. Furthermore, chronic MLL may lead to cosmetic deformities [1]. Late-onset breast deformities, which may have been chronic MLLs, have been reported in women with seatbelt injuries [7]. In our case, surgical removal was performed early given these risks, and favorable results were achieved.

## Conclusions

We described a case of a patient who underwent surgical removal of the MLL in the breast during the acute phase, leading to favorable outcomes. In chronic cases, recurrences that are difficult to treat have been reported. Additionally, MLLs may result in cosmetic deformities. This report highlights the importance of early detection and treatment of MLL in female breasts.
